# Methylation Patterns and Chromatin Accessibility in Neuroendocrine Lung Cancer

**DOI:** 10.3390/cancers12082003

**Published:** 2020-07-22

**Authors:** Elsa Arbajian, Mattias Aine, Anna Karlsson, Johan Vallon-Christersson, Hans Brunnström, Josef Davidsson, Sofie Mohlin, Maria Planck, Johan Staaf

**Affiliations:** 1 Division of Oncology, Department of Clinical Sciences Lund, Lund University, Medicon Village, SE 22381 Lund, Sweden; elsa.arbajian@med.lu.se (E.A.); mattias.aine@med.lu.se (M.A.); anna_f.karlsson@med.lu.se (A.K.); johan.vallon-christersson@med.lu.se (J.V.-C.); maria.planck@med.lu.se (M.P.); 2 Division of Molecular Hematology, Department of Laboratory Medicine, Faculty of Medicine, Lund University, SE 22184 Lund, Sweden; josef.davidsson@med.lu.se; 3 Division of Pathology, Department of Clinical Sciences Lund, Lund University, SE 22100 Lund, Sweden; hans.brunnstrom@med.lu.se; 4 Division of Genetics and Pathology, Department of Laboratory Medicine, Region Skåne, SE 22185 Lund, Sweden; 5 Division of Translational Cancer Research, Department of Laboratory Medicine, Lund University, Medicon Village, SE 22381 Lund, Sweden; sofie.mohlin@med.lu.se; 6 Division of Pediatrics, Department of Clinical Sciences Lund, Lund University, SE 22185 Lund, Sweden; 7 Department of Respiratory Medicine and Allergology, Skåne University Hospital, SE 22185 Lund, Sweden

**Keywords:** ATAC-seq, open chromatin, lung cancer, DNA methylation, neuroendocrine lung cancer

## Abstract

Lung cancer is the worldwide leading cause of death from cancer. Epigenetic modifications such as methylation and changes in chromatin accessibility are major gene regulatory mechanisms involved in tumorigenesis and cellular lineage commitment. We aimed to characterize these processes in the context of neuroendocrine (NE) lung cancer. Illumina 450K DNA methylation data were collected for 1407 lung cancers including 27 NE tumors. NE differentially methylated regions (NE-DMRs) were identified and correlated with gene expression data for 151 lung cancers and 31 human tissue entities from the Genotype-Tissue Expression (GTEx) consortium. Assay for transposase-accessible chromatin sequencing (ATAC-seq) and RNA sequencing (RNA-seq) were performed on eight lung cancer cell lines, including three NE cell lines, to identify neuroendocrine specific gene regulatory elements. We identified DMRs with methylation patterns associated with differential gene expression and an NE tumor phenotype. DMR-associated genes could further be split into six functional modules, including one highly specific gene module for NE lung cancer showing high expression in both normal and malignant brain tissue. The regulatory potential of NE-DMRs was further validated in vitro using paired ATAC- and RNA-seq and revealed both proximal and distal regulatory elements of canonical NE-marker genes such as CHGA, NCAM1, INSM1, as well as a number of novel candidate markers of NE lung cancer. Using multilevel genomic analyses of both tumor bulk tissue and lung cancer cell lines, we identified a large catalogue of gene regulatory elements related to the NE phenotype of lung cancer.

## 1. Introduction

Lung cancer is broadly divided into different histological subtypes, including adenocarcinoma (AC), squamous cell carcinoma (SqCC), large cell carcinoma (LCC), large cell neuroendocrine carcinoma (LCNEC), and small cell lung cancer (SCLC). Correct diagnosis of lung cancer histology is clinically important as it can dictate treatment (e.g., chemotherapy) in both early and late stage disease [1,2], and is strongly associated with the occurrence of actionable oncogenic alterations such as *EGFR* mutations and *ALK* gene fusions [3]. On the basis of extensive molecular characterization, it is now firmly established that the main histological subtypes represent distinct biological and molecular entities of lung cancer at the DNA, RNA, and protein levels [4,5,6,7,8].

In the most recent WHO guidelines [9], LCNEC and SCLC form a neuroendocrine (NE) subgroup which shares molecular similarities and, increasingly, also treatment strategies [4,8,10,11,12]. Considering the distinct genomic and histopathological phenotypes of the histological subgroups, it has been hypothesized that NE tumors develop from a cell of origin distinct from that of non-NE tumors [3,13]. Cellular differentiation states are regulated by different mechanisms, including epigenetic modifications. The latter constitute a dynamic layer of regulation involved in all aspects of cellular development and differentiation. In lung cancer, only a limited number of studies analyzing global DNA methylation and chromatin patterns across different histological subtypes have been reported [14,15,16,17,18]. Although few, these studies have corroborated that histological subtypes, in addition to having distinct transcriptional patterns, also carry defining and specific epigenetic patterns, with NE tumors typically being markedly different [8,14]. However, no directed global analyses of specific epigenetic patterns driving neuroendocrine transcriptional programs have been reported to date. 

In the current study, we aimed to define the epigenetic landscape of NE lung tumors and to identify putative regulatory elements of the NE phenotype of lung cancer by using global DNA methylation analysis of 1407 lung cancers of all histological subtypes and combined assay for transposase-accessible chromatin sequencing (ATAC-seq) and RNA sequencing (RNA-seq) of eight cell lines representative of the major histological subtypes. On the basis of the integrative analyses of DNA methylation patterns and gene expression data in both lung cancer and normal human tissue, we were able to define NE-specific gene regulatory elements. The epigenetic state of these were highly predictive of proximal gene expression, and associated genes could be mapped onto six distinct modules representing both tumor intrinsic and extrinsic processes. Importantly, one of the identified modules constituted a neural 178-gene module highly expressed in brain tissue with the capacity to accurately identify tumors of the NE lineage. Then, we demonstrated by combined ATAC- and RNA-seq that NE specific differentially methylated regions (DMRs) overlap regions of differentially accessible chromatin and that correlations observed in primary tumors between DNA methylation and gene expression could also be captured in an in-vitro context. Predictably, we showed that NE-associated gene regulatory elements were enriched for genes involved in neural differentiation and development, and identified a number of high-confidence regulatory elements controlling the expression of canonical NE-marker genes such as chromogranin A (*CHGA*), neural cell adhesion molecule (*NCAM1*/*CD56*), and the insulinoma-associated 1 (*INSM1*) gene. Taken together, this study defines a compendium of epigenetic regulators of the NE phenotype of lung cancer which serves as a foundation for future investigations into the gene regulatory networks involved in the development and progression of NE lung cancer. 

## 2. Results

**Figure 1:**
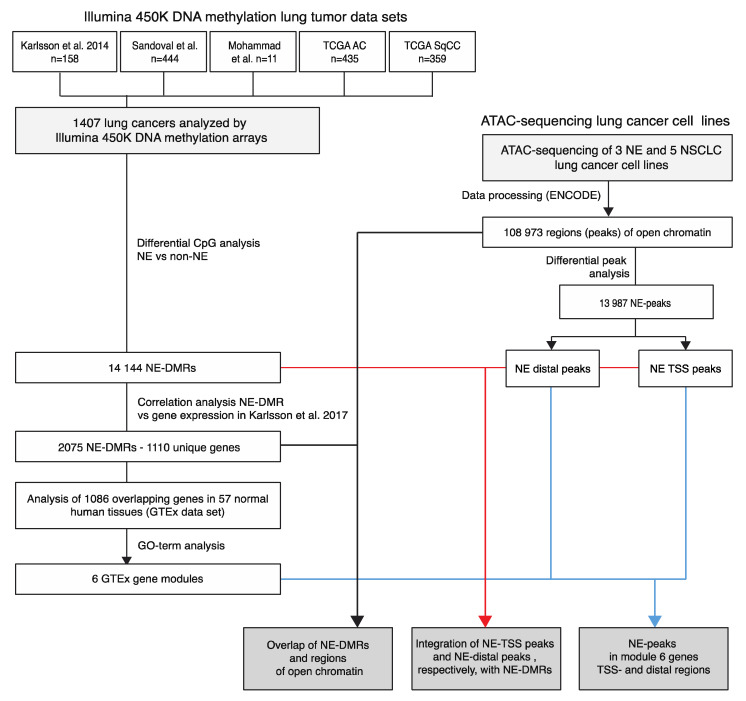
Study scheme. Cohorts listed refers to Karlsson et al. [14], Sandoval et al. [15], cancer genome atlas (TCGA) adenocarcinoma (AC) [5], TCGA squamous cell carcinoma (SqCC) [7] for Illumina 450K methylation data. Gene expression cohorts involved refers to Karlsson et al. [8]. TSS, transcription start site; NE, neuroendocrine; DMR, differentially methylated region; NSCLC, non-small cell lung cancer.

The outline of the study is described in Figure 1.

### 2.1. Differentially Methylated Regions in Neuroendocrine Lung Cancer Correlated with Gene Expression

After filtering, a final dataset interrogating 459,790 genomic positions for the 1407 tumors profiled by DNA methylation arrays remained. A total of 16,063 differentially methylated probes (CpGs) was observed between NE tumors versus normal lung and non-NE tumors (Mann–Whitney U test, Bonferroni *p* < 0.01, absolute difference in median methylation >0.1 in both comparisons). To form the final set of NE differentially methylated regions (NE-DMRs), we assigned each significant probe a 101 bp genomic window and merged probes with overlapping windows (n = 3214 probes mapping to 1295 regions) for each sample. This yielded a final set of 14,144 NE-DMRs. 

To investigate the association with gene expression, we correlated NE-DMRs with matched transcriptional data from Karlsson et al. [8]. Using a 1 Mb annotation window for each of the 14,144 NE-DMRs, NE-DMRs were on average linked to 9.6 unique genes for which gene expression was available. A total of 663 NE-DMRs could not be linked to any gene for which expression data were available. For the 13,481 NE-DMRs which could be linked to gene expression data, we calculated the Pearson correlation between DNA methylation beta and mRNA expression resulting in a total of 135,250 correlation coefficients. We used the 98th percentile of the absolute *p*-value distribution as a significance cut-off (r more extreme than +/−0.496), a more stringent choice than that obtained by Bonferroni correction (approximate correlation cut-off = 0.4). This yielded 1306 significant positive and 1399 significant negative correlation coefficients between NE-DMRs and genes. From the way the test was set up, a single NE-DMR was allowed to correlate with the expression of multiple genes, and a given gene could be correlated to multiple NE-DMRs. In total, the 2705 significant correlation coefficients mapped to 2075 unique DMRs and 1110 unique genes (Figure 1 and Supplementary Table S1).

### 2.2. NE-DMR Genes Expression in Normal Tissues Defines Six Transcriptional Programs

**Figure 2:**
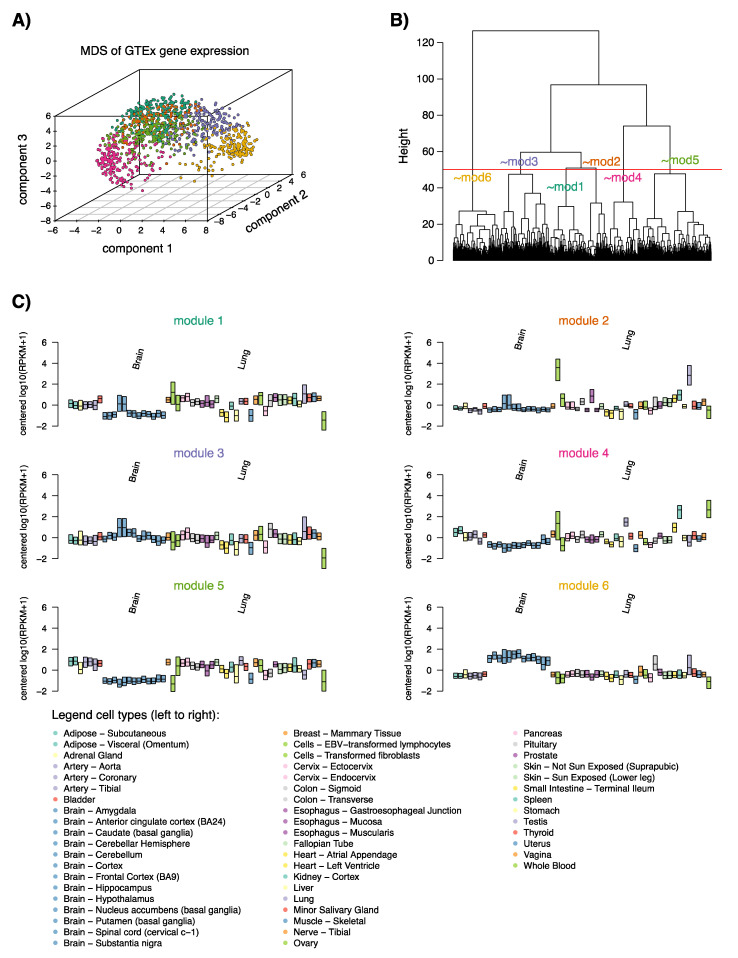
Deriving Genotype-Tissue Expression (GTEx) gene modules. (**A**) Multidimensional scaling (MDS) of gene expression of 1086 genes that correlate between NE-DMRs and gene expression in expression data from 53 GTEx tissue samples corresponding to 31 tissue types and cell lines. Prior to MDS, expression was transformed using log10 (RPKM + 1) and mean-centered. For genes with multiple entries, the entry with the highest standard deviation entry was chosen. The different colors represent the modules as defined in (B); (**B**) Definition of six gene modules based on hierarchical clustering. Modules 1–6 are referred to as follows: (i) early cell cycle/metabolism (n = 193 genes), (ii) proliferation (n = 133 genes), (iii) metabolic process/inconclusive (n = 165 genes), (iv) immune activation/infiltration (n = 197 genes), (v) angiogenesis/TGFb-SMAD (n = 220 genes), and (vi) neurodevelopment/brain (referred to as the neural module, n = 178 genes); (**C**) Module expression (average of standardized expression of included genes) across GTEx tissue types. Boxplots show the distribution of expression for respective module’s genes in tissue samples according to cell type legend.

To understand the transcriptional programs of NE-DMRs linked with gene expression in lung cancer, we investigated the expression of the 1110 unique genes in 53 non-cancer sample types corresponding to 31 human tissues and transformed fibroblasts and lymphocytes from the Genotype-Tissue Expression (GTEx) consortium. Of the 1110 genes, 1086 genes could be matched, and were analyzed for the presence of transcriptional gene modules using k-means clustering for dimensionality reduced gene expression data (see methods). Six gene modules were identified in the GTEx data (GTEx modules), showing transcriptional differences across tissue types (Figure 2). Gene ontology (GO-) term analysis identified the modules as related to (in some instances partly) the following: (i) early cell cycle/metabolism (n = 193 genes), (ii) proliferation (n = 133 genes), (iii) metabolic process/inconclusive (n = 165 genes), (iv) immune activation/infiltration (n = 197 genes), (v) angiogenesis/TGFb-SMAD-signaling (n = 220 genes), and (vi) neurodevelopment/brain (referred to as the neural module hereafter, n = 178 genes) (Supplementary Table S2). Notably, the neural GTEx signature (module 6) included three of the currently used diagnostic marker genes for NE classification, *CHGA*, *NCAM1* (*CD56*), and *INSM1,* but not *SYP*. GO-term analysis was also performed on NE-DMR correlated, downregulated genes in NE tumors (Supplementary Table S2). 

### 2.3. Characterization of the Six GTEx Modules in Lung Cancer Gene Expression Data

**Figure 3:**
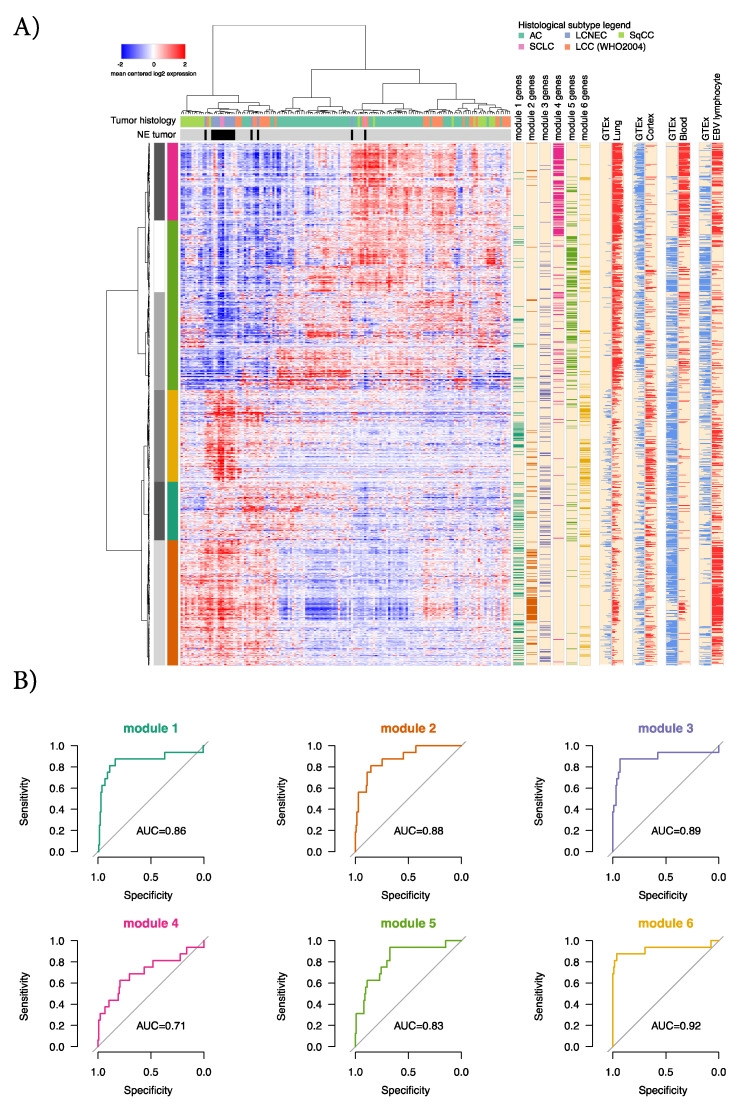
GTEx gene modules in lung cancer and tumor reclassification. (**A**) Gene expression clustering in 151 lung cancers (columns) from Karlsson et al. [8] based on genes (rows) in the six GTEx modules, using Pearson distance and Ward’s method. The horizontal column color bars above the expression heatmap indicate lung cancer histology and NE-status, respectively. The grey-white panel to the right of the row dendrogram shows six gene clusters derived by cutting the dendrogram at the six-group level. The next color panel shows enrichment of GTEx modules within the six data-derived gene clusters (all *p* < 0.001, Fishers exact test). The vertical color bars to the right of the heatmap indicates which module each gene belongs to. The rightmost four vertical panels show rank scores for each gene within the indicated cell type. For these, the bars extending to the leftmost edge signify lowest relative expression across 53 GTEx cell types, the bars extending to the rightmost edge signify highest relative expression; (**B**) Classification results for the different gene modules in predicting NE status in the 151 cases.

**Figure 4:**
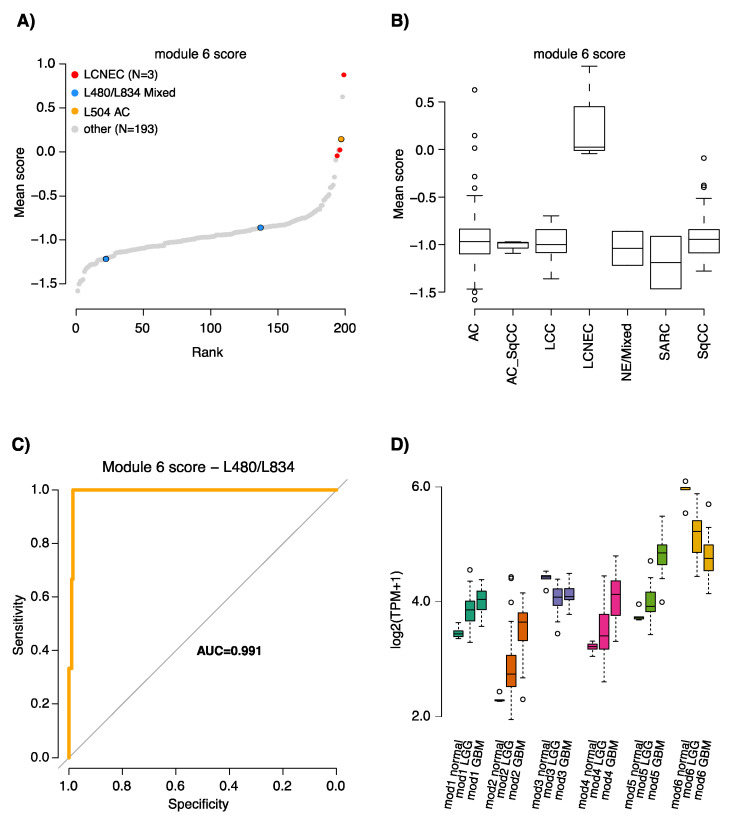
Validation and expression of the GTEx neural gene module in tumor data and normal brain, low-grade glioma, and glioblastoma. Analysis of the GTEx neural gene module in RNA-seq data from Djureinovic et al. [19]. (**A**) Sorted gene module scores in the dataset. Legend shows sample annotations for large cell neuroendocrine carcinoma (LCNEC) tumors confirmed as LCNEC in re-review [20], two tumors with mixed histological subtypes where only the adenocarcinoma component was RNA sequenced, and one original adenocarcinoma case (L504) with immunohistochemistry expression of a diagnostic NE marker (*CHGA*) (see [20] for details); (**B**) Gene module scores stratified by tumor histological subtypes. LCC, large cell carcinoma; AC_SqCC, adenosquamous; SARC, sarcomatoid; (**C**) ROC curve showing classification results for the gene module when not including tumors L480 and L834 of mixed NE/non-NE histology; (**D**) Expression of the neural GTEx gene module in normal brain tissue (n = 5), low-grade glioma (LGG, n = 25), and high-grade (n = 25) glioblastoma multiforme (GBM).

To assess the gene modules derived from the GTEx normal tissue collection, we clustered the 151 lung tumors from Karlsson et al. [8], finding that coexpression of the individual module genes was largely conserved in lung cancer. This indicated that normal gene expression circuits could be co-opted in the process of malignant transformation (Figure 3A). To assess the gene modules performance for NE prediction, we calculated the mean expression scores for each lung cancer sample and gene module from row-standardized expression values. Next, the specificity of gene module expression was evaluated using receiver-operating characteristic (ROC) curves, showing that the neural GTEx signature (module 6) was highly predictive of a NE phenotype (AUC = 0.92) (Figure 3B). We confirmed the latter result in the dataset reported by Djureinovic et al. [19] that comprises RNA sequencing data from 199 lung tumors, showing the specificity and association of module 6 with an NE phenotype (Figure 4A–C). When the gene signatures were applied to 25 glioblastoma multiforme (GBM) tumors, 25 low-grade gliomas, and five normal brain tissue specimens from the TCGA study, it again confirmed the elevated expression of the neural gene module in normal brain tissue, low-grade glioma, and high-grade GBMs (Figure 4D). 

### 2.4. Assay for Transposase-Accessible Chromatin Sequencing (ATAC-Seq) Identifies Regions of Open Chromatin Associated with NE Histology

We performed ATAC-seq and complementary RNA-seq on five NSCLC and three NE lung cancer cell lines. After initial processing and filtering, a total of 108,973 regions (peaks) of open chromatin remained, and of these peaks, 13,987 peaks showed differential presence in NE vs. NSCLC cell lines (FDR adjusted Wald test, *p* < 0.05, Supplementary Table S3). 

**Figure 5:**
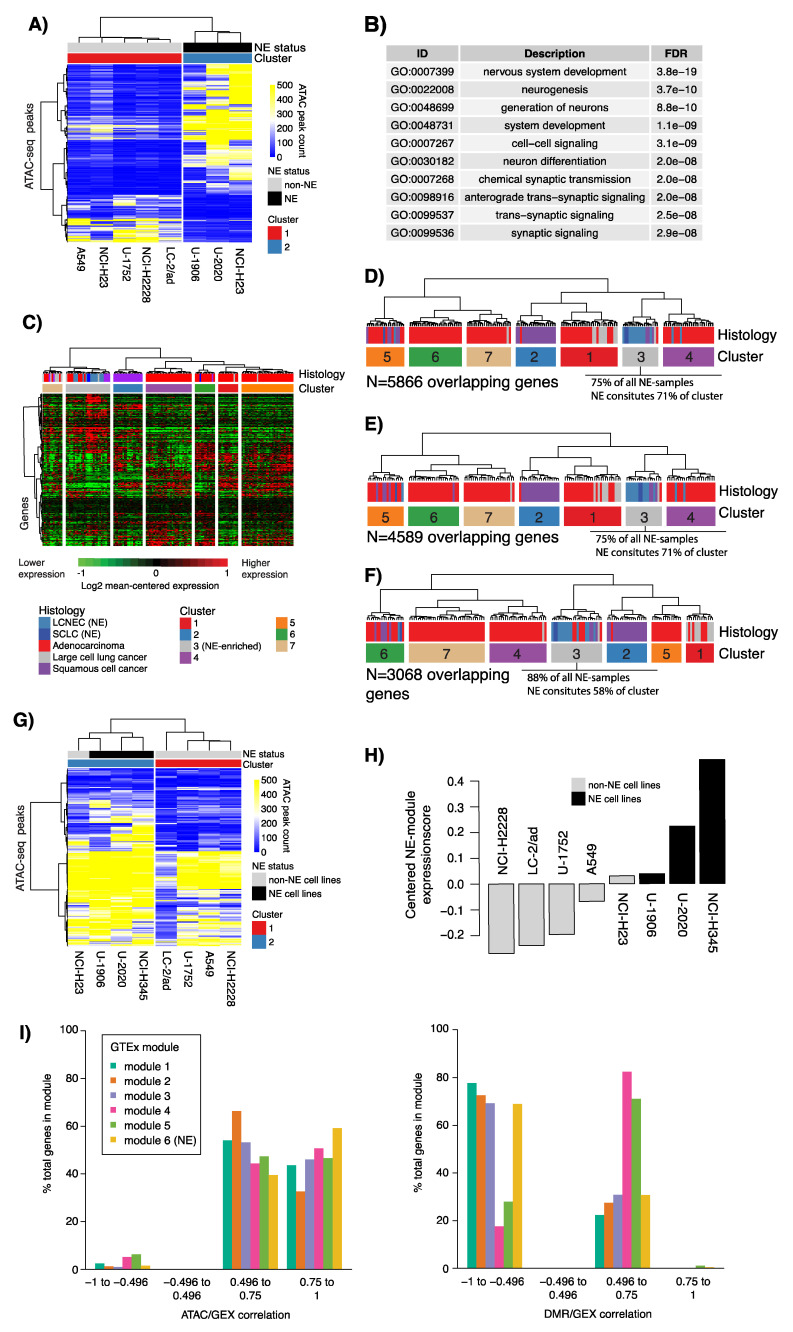
Assay for transposase-accessible chromatin sequencing (ATAC-seq) of NE and non-NE lung cancer cell lines and integration with tumor derived DMRs. (**A**) Clustering of 564 NE-associated TSS ATAC-seq peaks with absolute Pearson correlation to expression >0.7 in eight lung cancer cell lines using Euclidean distance and ward linkage; (**B**) Top 10 gene ontology terms for genes associated with the 733 NE-associated TSS ATAC-seq peaks; (**C**) Clustering of 338 genes matching the 540 unique genes from the 564 TSS peaks in A in bulk tumor tissue gene expression data from 151 lung cancers reported by Karlsson et al. [8]. Genes (rows) and samples (columns) were clustered using Pearson correlation and ward.D linkage. Expression data were in the form of mean-centered log2 transformed intensity values; (**D**) Clustering of 5866 genes matching 8729 distal (10 Kbp) ATAC-seq peaks defined by differential peak count between NE and non-NE cell lines and correlated with RNA-seq for genes in a ±1 Mbp window in the 151 tumor tissues [8]. Genes (rows) and samples (columns) were clustered using Pearson correlation and ward.D linkage. Expression data were in the form of mean-centered log2 transformed intensity values. Only the cluster tree is shown; (**E**) Similar analysis as in (D) but now for genes within a ±500 kbp window. Only the cluster tree is shown; (**F**) Similar analysis as in (D) but now for genes within a ±200 kbp window. Only the cluster tree is shown; (**G**) Clustering of 197 TSS ATAC-seq peaks matched to genes in the neural GTEx module (module 6) in the eight lung cancer cell lines using Euclidean distance and ward linkage; (**H**) Average expression of the neural GTEx module in the eight cell lines based on mean-centered log2 transformed expression data; (**I**) Fraction genes in each of the six GTEx gene modules with a specific range of Pearson correlation between ATAC-seq data and RNA-seq (GEX) in cell lines, and DMR beta values and gene expression in tumors of correlation.

Among the 13,987 peaks, 977 were associated with transcription start site (TSS +/− 1000 bp) in 923 unique protein coding genes. The 977 TSS peaks showed high Pearson correlation values with corresponding RNA sequencing data for matched genes (mean of positive correlations = 0.73). Filtering of the 977 TSS peaks versus gene expression correlation (absolute Pearson correlation > 0.7) resulted in 564 peaks in 540 unique protein coding genes that, as expected, could separate NE from NSCLC cell lines using hierarchical clustering (Figure 5A). Gene ontology analysis of these 540 genes revealed overrepresentation of neural gene ontology terms, consistent with the finding of a tumor expressed neural GTEx module (Figure 5B and Supplementary Table S4). Prototypical diagnostic NE marker genes were included, such as *CHGA*, *NCAM1* (*CD56*), and *INSM1*. Hierarchical clustering using 338 (of 540) matching genes in the cohort of 151 lung tumors from Karlsson et al. [8] formed one gene expression cluster that included 94% of all NE-tumors (Figure 5C). In addition, the clustering stratified NSCLC tumors grossly by histological subtype. 

Next, we next investigated ATAC-seq peaks statistically different between NE and non-NE cell lines but distal of TSSs (10 kbp). For each peak, we identified, within a ±1 Mbp window, all genes with a Pearson correlation >0.7 between RNA-seq and ATAC-seq peak counts (n = 7874 genes, n = 9720 peaks), with the hypothesis that peak gene pairs could represent putative distal regulatory elements. Hierarchical clustering of matching genes (n = 5866) in the 151-sample tumor cohort almost perfectly discerned the histological subtypes (Figure 5D). Similar results were obtained for window sizes of ±500 kbp (n = 7647 peaks and n = 6139 genes) and ±200 kbp (n = 5180 peaks and n = 4108 genes) (Figure 5E,F).

Finally, to investigate whether the neural GTEx module (module 6) accounted for the NE component of the NE cell lines, we extracted both ATAC-seq peaks in transcription start site regions (TSS +/− 1000 bp) of module six genes and ATAC-seq peaks distal to the TSS of these genes. Clustering of the TSS ATAC-seq peaks separated NE cell lines from non-NE cell lines, with the exception of one NSCLC cell line (H23) (Figure 5G), which showed some expression of neural module genes by RNA-sequencing (Figure 5H). Clustering of distal ATAC-seq peaks to the neural module genes (n = 1158, annotation window TSS +/− 500 kb) separated two out of three NE cell lines into a single cluster, while the U-1906 NE cell line clustered with remaining samples (Supplementary Figure S1).

### 2.5. ATAC-Seq Supports Epigenetic Regulation of NE-DMR Genes

Tumor-based DNA methylation findings were integrated with cell line ATAC-seq by subanalysis of the 1110 unique genes that showed a significant gene expression correlation with NE-DMR methylation. Of these, 827 (75%) also showed significant Pearson correlation between RNA expression and ATAC-seq signal in the eight cell lines. Of the 827 genes, 585 (71%) had ATAC-seq peaks in the TSS region (TSS +/− 1000 bp), including diagnostic NE marker genes such as *CHGA*, *NCAM1* (*CD56*), and *INSM1*. Gene ontology enrichment analysis of the 585 genes showed overrepresentation of multiple gene ontology terms associated with cell proliferation, biosynthesis, metabolic processes, but also a number of cell development and neural associated terms (Supplementary Table S4). Notably, when analyzing the overall pattern of correlations between tumor DNA methylation and gene expression for the genes with ATAC-seq peaks in the promoter region, we found that the neural GTEx module genes showed the strongest positive correlations in the ATAC-seq data and negative correlations in the tumor methylation data (Figure 5I). Figure 5I also illustrates that two GTEx modules (IV, immune activation/infiltration and V, angiogenesis/TGFb-SMAD) show predominantly positive DMR-GEX correlation in tumors. It is conceivable that these modules represent the contribution of non-tumor signal, because the data were generated from bulk cancer tissue.

### 2.6. Assessing the Regulatory Potential of Tumor Derived NE-DMRs in ATAC-Seq Data

To validate the regulatory potential of tumor-derived DMRs in ATAC-seq data, first, we adjusted the 14,144 DMRs to a consensus window size of 500 bp. Of the 14,144 DMRs, 33.5% overlapped with an ATAC-seq peak. Of the 2075 DMRs with significant correlation to gene expression data, 45.4% overlapped (n = 942), indicating that a large proportion of NE-DMRs represent bona fide regulatory elements.

Conversely, of all 13,987 NE-associated ATAC-seq peaks, 5.8% (819/13,987) overlapped with a DMR region. Similar overlap frequencies were seen for the 10 kbp distal peaks irrespective of window size (±1 Mbp window 6.5% overlap, ±500 kbp window 6.8% overlap, and ±200 kbp window 7.2% overlap). In contrast, for the 564 TSS peaks, 13.7% overlapped with a DMR. This larger overlap was likely due to bias in CpG probe positioning on the Illumina platform. Nonetheless, our results indicate that high correlations between DNA methylation and gene expression in bulk tumor material can be used to capture putative regulatory associations.

With respect to distal regulatory interactions, integration of bulk tumor methylation sequencing and cell line ATAC-seq data supported the existence of several candidate DMRs involved in the regulation of NE-specific gene expression (Supplementary Table S5).

**Figure 6:**
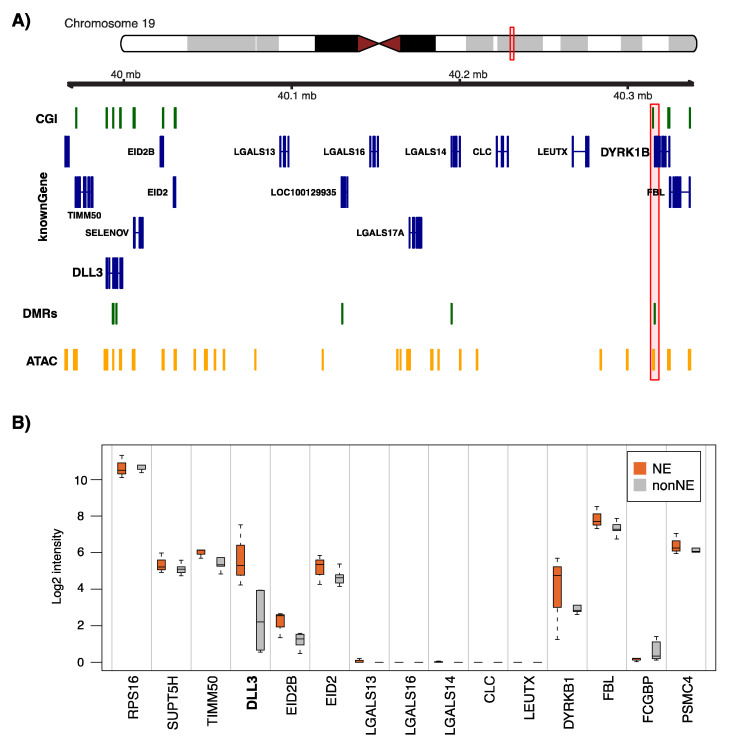
Example of distal ATAC-seq peaks overlapping with tumor DMR in *DLL3*. (**A**) Genomic location of NE-associated ATAC-seq peaks and tumor DMRs surrounding the *DLL3* gene. The position of the distal ATAC-seq peak (with Pearson correlation to RNA expression in cell lines >0.7) is highlighted; (**B**) Gene expression of genes surrounding the *DLL3* gene and the distal ATAC-seq peak in tumors from Karlsson et al. [8] stratified by NE status.

Figure 6A illustrates a putative distal regulatory element for the established NE-gene *DLL3* [21]. The DMR is situated within the *DYRK1B* gene, approximately 200 kbp distal to the *DLL3* TSS, and is significantly correlated with *DLL3*, but not *DYRK1B*, gene expression in both primary tumor samples and cell lines. Expression analysis of genes in the region surrounding *DLL3* demonstrates the putative demarcation of a regulatory domain by the aforementioned NE-DMR with markedly reduced expression of several genes between *DLL3* and the ATAC-supported distal NE-DMR, but with higher expression of genes both upstream and downstream of the silent genomic segment consistent with a putative looping-type interaction (Figure 6B). 

## 3. Discussion

In this study, we investigated DNA methylation patterns and regions of open chromatin specific for NE versus non-NE lung cancer. On the basis of the strong evidence of the histological subtypes representing distinct molecular entities [4,8,10,14,15,17], we found that epigenetic changes specific for NE cancer were pervasive, and that a substantial proportion of these could be gene regulatory in nature. 

To test our hypothesis, we used high-resolution DNA methylation profiles of >1400 lung cancers of all histological types, allowing us to identify 14,144 NE-DMRs. These DMRs, on average, were linked to 9.6 unique genes per DMR, of which 2075 DMRs and 1110 unique genes showed significant correlations between DNA methylation and gene expression in lung cancer. To understand the composition of these genes, i.e., to determine if they grouped in biological subsets, we analyzed the expression of these genes broadly in 29 non-malignant human tissue types, identifying six modules of tightly regulated genes. One of these modules appeared as a neural gene module, based on functional annotation and high expression in normal, as well as malignant, brain tissue. This module was capable of predicting NE lung tumors with high accuracy and included three currently used diagnostic marker genes for NE classification (*INSM1*, *NCAM1,* and *CHGA*) but not the fourth gene, synaptophysin (*SYP),* since this gene was not correlated to any of the derived NE-DMRs. However, *SYP* did have two distal regions of open chromatin highly correlated to the expression of the gene and significantly enriched in NE cell lines as compared with NSCLC. In support of the tumor-based analyses, regions of open chromatin in the TSS regions of the neural module genes could differentiate ATAC sequenced NE from NSCLC cell lines, with the exception of H23, an NSCLC cell line showing some expression of neural module genes. 

While the significance of the neural gene module has a conceptually simple link to NE lung cancer, we identified five other gene modules from a diverse set of normal human tissue types (Figure 2). Some of these also classified NE tumors with fairly high accuracy, however, they appeared less distinct for NE when considering their gene expression patterns in lung cancer in general (Figure 3A,B). For instance, genes in module two (associated with proliferation) show high expression also in subsets of SqCC, AC, and WHO 2004 LCC cases, while genes in module four (associated with immune activation/infiltration) are low in expression in NE cancers but also in other subsets of SqCC, LCC, and AC consistent with previous observations [8]. Thus, these gene modules could represent a difference in cellular composition with respect to non-malignant cells between NE tumors and non-NE tumors in general, with NE cases being generally more proliferative and characterized by a more dense growth pattern [22]. One indication of this could be that two gene modules showed mainly positive correlation between DNA methylation and gene expression data, in contrast to the expected negative correlation (Figure 5I). 

To extend our analyses of NE specific transcription, we performed ATAC- and RNA-seq of the NE and NSCLC cell lines. Despite the heterogeneity of U-1906 and H23 (NE and NSCLC cell lines, respectively), both showing moderate expression of neural module genes at RNA sequencing, the ATAC-seq data provided robust evidence for NE specific regions of open chromatin. In fact, NE-associated TSS regions of open chromatin were strongly enriched for neural associated genes, and TSS associated genes were able to recapitulate the major lung cancer histological groups including NE tumors in bulk tumor gene expression data. The latter was true also in bulk tumor tissue for genes associated with distal ATAC-seq peaks (Figure 5D–F). Approximately 14% of TSS peaks overlapped with a tumor DNA methylation DMR as compared with approximately 7% of NE-associated peaks distal of TSSs that overlapped with tumor DMRs. The limited overlaps were likely due to the platform design of the Illumina Human Methylation 450K Beadchips, focusing on CpG islands and promoters as compared with the genome-wide coverage of ATAC-seq, limiting an unbiased “validation” of ATAC-seq peaks from the in vitro analysis in tumor tissue. The latter especially applies to distal ATAC-seq peaks (representing putative distal gene regulatory elements). Nevertheless, overlap analysis identified well-established NE-associated genes such as *DLL3* and its upstream regulator, achaete-scute complex-like 1 gene (*ASCL1*), as having distal ATAC-seq peaks and overlapping a tumor DMR in bulk tissue analysis. *ASCL1* is a transcription factor critical for development of pulmonary neuroendocrine cells in the developing lung, with *DLL3* as a downstream target [23]. These form the ASCL1–DLL3–Notch1 pathway which is frequently perturbed in SCLC [24,25]. Analysis of expression patterns surrounding the *DLL3* TSS and the distal peak region showed no expression of genes in between the TSS and distal region, consistent with a chromatin-loop mediating the regulatory function (Figure 6B). Although the analyses performed in the current study did not provide functional evidence for epigenetic regulation of, for example, *DLL3*, *NCAM1*, or *INSM1*, in NE lung cancers, it has been reported in hepatocellular carcinoma that *DLL3* appears to be regulated by promoter methylation (silenced by hypermethylation), and that, for example, 5-Aza-2′-deoxycytidine treatment of cancer cell lines could reactivate expression of the gene [26,27]. Clearly, additional functional studies, coupled with genome-wide omics characterization, are needed that also consider, for instance, the NE transcription and differentiation factors. Together, the results in the current study highlight the hypothesis generating potential of multilevel genomic characterization that may facilitate the identification of novel druggable targets in SCLC based on further functional studies. 

Finally, we investigated the potential of our data in addressing the need for more specific and sensitive clinical NE markers [28]. Given the specificity of module 6 genes to neural tissues, we further identified a subset with both regions of open chromatin highly positively correlated to gene expression in the NE cell lines and an associated NE-DMR with significant negative correlation to gene expression in bulk tissue, and thus proposed a set of candidate genes to be investigated for their potential as clinical NE markers (Supplementary Table S5). Although definitive supportive data are currently lacking, future experiments utilizing chromatin conformation capture technology [29] on cell lines representative of the major LC histological subtypes have the potential to greatly expand our understanding of the gene-regulatory landscape of neuroendocrine LC. 

## 4. Materials and Methods

### 4.1. Patient Cohorts

A DNA methylation dataset of 1407 lung tumors and 88 histologically normal lung samples was compiled from previously published research studies and the TCGA data portal (obtained as level 3 data) [5,7,14,15,30], or was generated specifically for this study (n = 36 tumors). All cases were analyzed using the Illumina methylation 450K platform [31]. Sample annotations, including tumor histology based on the WHO 2004 classification, were collected from each study for previously reported data. For the 36 new tumors, gene expression data have been previously reported along with clinical data in Karlsson et al. [8] but not DNA methylation profiles. The combined set of new tumors and previously reported DNA methylation analyzed cases from [14] that are also included in [8] is referred to as Karlsson et al. (Figure 1). Histological subtypes (five LCNEC and 31 LCC) for the new tumors were determined by a lung cancer pathologist based on the WHO 2004 classification scheme that was the current guideline at the time of data generation. The final set consisted of 840 AC, 504 SqCC, 14 SCLC, 13 LCNEC, and 36 tumors of other histological subtypes (e.g., LCC).

### 4.2. DNA Methylation Analyses

DNA methylation profiling using Illumina Human Methylation 450K beadchips was performed for 36 cases specifically for this study at the Centre for Translational Genomics, Lund University and Clinical Genomics Lund, SciLifeLab using protocols and methods as previously described [14]. DNA methylation data were uniformly processed using methods described previously [14,32], resulting in a final processed dataset interrogating 473,864 autosomal genomic positions. An initial data filtering was performed to remove probes with >5% missing values in tumors classified as NE, non-NE, or normal lung tissue. Next, we removed probes with zero standard deviation across the respective groups. In total 14,074 probes were eliminated by these steps and resulted in a final dataset interrogating 459,790 genomic positions.

The Mann–Whitney U test (Bonferroni adjusted, *p* < 0.01) comparing NE tumors versus normal lung and non-NE, respectively, was used to identify positions in the genome associated with NE lung cancer. Differentially methylated regions (NE-DMRs) were formed by assigning each significant probe a 101 bp genomic window and merging probes with overlapping windows by their median value. Each NE-DMR was annotated to genomic transcripts using the UCSC hg19 “known gene” database contained in the Bioconductor package “TxDb.Hsapiens.UCSC.hg19.knownGene” (db-version 1.1). Transcripts were further matched to gene symbols using the Bioconductor package “org.Hs.eg.db” (db-version 2.1) and each NE-DMR was annotated with transcript, promoter, and exon overlaps. Illumina CpG ID’s were carried over to the NE-DMRs and CpG island or shore overlaps were assigned using the UCSC genome browser RefCgi-track (hg19 data freeze). To explore putative distal regulatory associations we linked each NE-DMR to TSSs inside of 500 kb up- or downstream from the NE-DMR-midpoint. The choice of 500 kb was motivated by similar analyses performed by the FANTOM [33] and cancer genome atlas (TCGA) [18] consortia. All overlap calculations were performed using the Bioconductor package GenomicRanges [34] in the R-programming environment [35]. Analyses are further detailed in Supplementary Methods.

### 4.3. Gene Expression Analyses

Preprocessed and normalized gene expression data derived using Illumina HT12 v4 arrays were available for 151 of the tumors included in the methylation dataset [8]. The most variable probe was selected for each unique gene resulting in a final dataset with expression for 20,440 unique genes. For matching to NE-DMR annotations we used Entrez identifiers. 

To analyze the expression of correlated genes from the NE-DMR analysis in normal tissues, average gene expression data for 53 sample types corresponding to 31 human tissues and transformed fibroblasts and lymphocytes were obtained from the GTEx consortium (www.gtexportal.org, v6p data freeze). The dimensionality of the variation patterns of the genes in the GTEx data was reduced using multidimensional scaling (MDS, R function cmdscale, 3 dimensions) of the row-standardized expression matrix and visualized in 3 dimensions in R (R package scatterplot3d). Expression modules were derived using k-means clustering of the MDS output (R function k-means, K = 6). Agglomerative hierarchical clustering using Euclidean distance and Ward’s method (R function hclust with method “ward.D2”) was used as a complementary approach to evaluate the six expression modules. Statistical overrepresentation GO-term analysis was performed using the Panther web tool (www.pantherdb.org).

Mean expression scores were derived for each LC sample and gene module from row-standardized expression values. Gene module expression scores by histological subtype were visualized using boxplots and the specificity of module gene expression was evaluated using receiver operating characteristic (ROC) curves in R (function roc in package pROC). Gene module expression was further assessed in RNA sequencing data from Djureinovic et al. [19] including 199 lung tumors of mixed histological subtypes. The expression data (FPKM) were offset by 0.5 and log2 transformed prior to module score calculations. Original pathological assessments for the dataset were updated with data from Karlsson et al. [20] who reclassified two NE samples (L480/L834) as mixed histology and one adenocarcinoma (L504) as having a NE component.

To assess NE-DMR methylation patterns and gene expression correlations in brain-derived samples, we obtained 450K and RNA-seq data from the Genomic Data Commons (GDC) portal [36] for 25 glioblastoma multiforme (GBM) and 25 low-grade glioma (LGG), as well as for normal brain samples (five with RNA sequencing and two with 450K data). Analyses are further detailed in Supplementary Methods.

### 4.4. ATAC- and RNA-Seq of NSCLC and NE Cell Lines

ATAC-seq was performed on the following eight cell lines: five NSCLC, NCI-H2228 (ATCC Cat# CRL-5935 RRID:CVCL_1543), LC-2/ad (ECACC Cat# 94072247 RRID:CVCL_1373), U-1752 (RRID:CVCL_0565) [37], A549 (ATCC Cat# CCL-185 RRID:CVCL_0023), NCI-H23 (ATCC Cat# CRL-5800 RRID:CVCL_1547); and three SCLC/NE, U-1906 (RRID:CVCL_D075) [37], U-2020 (RRID:CVCL_D076) [37], and NCI-H345 (ATCC Cat# HTB-180 RRID:CVCL_1558). All cell lines were grown as two replicates, according to standard protocols (Supplementary Methods). Libraries were prepared from 100,000 to 150,000 cells per replicate according to the Omni-ATAC protocol [38], with minor adjustments, i.e., the final spin column purification step for removal of primer-dimers and large fragments was replaced with double-sided AMPure XP bead purification. Then, libraries were sequenced on a NextSeq 500 (Illumina) with paired end reads of 80 bp. Data analysis was performed according to recent ENCODE guidelines, with minor changes, as outlined (Supplementary Methods), and produced a set of high confidence peaks for each cell line. Using the GenomicRanges [22], SoGGi (version 1.18.0), and Rsubread (doi: 10.1093/nar/gkz114) R packages, high confidence peaks form all cell lines were merged, overlapping peaks were reduced and the number of reads per peaks were counted. Normalization of peak counts and differential peak analysis in NE versus non-NE cell lines were performed with Deseq2 (doi:10.1186/s13059-014-0550-8). Then, the peaks were annotated to genomic transcripts using the UCSC hg19 “known gene” database contained in the Bioconductor package “TxDb.Hsapiens.UCSC.hg19.knownGene” (db-version 1.1), using the annotatePeak function in ChIPseeker. Transcripts were further matched to gene symbols using the Bioconductor package “org.Hs.eg.db” (db-version 2.1). Alternatively, peaks were annotated to all flanking genes within 2Mb, 1Mb, or 0.4 Mb windows centered on the peak midpoint (Supplementary Methods). RNA sequencing was performed on all cell lines as previously described to generate FPKM expression estimates [39], resulting in a dataset with expression for 17,897 unique protein coding genes matched to peaks through gene symbols. Gene expression data were correlated to matched peaks using the Pearson correlation.

### 4.5. Data Sharing

RNA-seq data (FPKM) and processed ATAC-seq data for NE and NSCLC cell lines are available through doi:10.17632/wmt6bvrwx8.1. DNA methylation data for additional NE tumors are available through GSE149521 at Gene Expression Omnibus.

## 5. Conclusions

We used a multilayered genomic approach to identify hundreds of putative causal regulators of the neuroendocrine tumor phenotype. Our approach identified regulatory elements capable of discriminating NE from non-NE lung cancer using two methodologically distinct approaches, bulk tumor analysis and in vitro experiments. Importantly, both layers of analysis identified largely the same set of genes as prominent features of the NE phenotype and even converged on a common set of regulatory elements with high correlations to proximal or distal gene expression. Future studies utilizing ATAC-seq optimized for frozen tissue [38] in combination with gene expression and DNA methylation analysis holds great potential for a more in-depth characterization of the pangenomic landscape of primary neuroendocrine lung cancer. While our current analyses were restricted to the question of the NE phenotype, a similar approach combining all the histological subtypes of lung cancer has the potential to provide new and more granular insights into the underlying biology of these tumor entities and facilitate the discovery of novel clinical biomarkers.

## Supplementary Materials

The following are available online at https://www.mdpi.com/2072-6694/12/8/2003/s1, Supplementary Figure S1: Clustering of distal ATAC-seq peaks for genes in the neural gene module. Of the 178 neural gene module genes, 1158 had an ATAC-seq peak within 1Mb window of their TSS. Clustering was performed using Euclidean distance and ward linkage, Supplementary Table S1: DMRs associated with NE in DNA methylation data, Supplementary Table S2: GTEx gene modules and GO-term analysis, Supplementary Table S3: NE-associated ATAC-seq peaks from analysis of lung cancer cell lines, Supplementary Table S4: Gene ontology analyses of genes derived from ATAC-seq alone or integration with DMRs, Supplementary Table S5: Putative marker genes of the neuroendocrine tumor phenotype. Supplementary Information. Detailed description of analysis steps.
